# Study on the transformation of nitrate nitrogen by manganese-catalyzed iron–carbon micro-electrolysis and microbial coupling

**DOI:** 10.1039/d4ra00377b

**Published:** 2024-04-04

**Authors:** Qiong Wan, Xiayin Li, Feng Wang, Guohong Yang, Kai Ju, Hongbin Jing, Kun Li, Peng He, Xinyan Zhang

**Affiliations:** a School of Architecture and Civil Engineering, Xi'an University of Science and Technology Xi'an 710054 China xyzhang2020@xust.edu.cn; b Shaanxi Water Group Water Ecology Comprehensive Development Co., Ltd 2206 Hongqi Road, Weiyang District Xi'an 710018 China

## Abstract

Nitrate-nitrogen pertains to the nitrogen component of the overall nitrate present in a given sample in order to reduce nitrate nitrogen pollution in water, nitrate nitrogen removal methods based on iron–carbon micro-electrolysis have become a key research focus. The process and mechanism of nitrate nitrogen removal by microbial coupling was comprehensively explored in a novel iron–carbon micro-electrolysis (ICME) system. In order to establish the transformation pathway of nitrate nitrogen in water, the transformation paths of nitrate nitrogen in water before and after coupling microorganisms in three groups of continuous flow reaction devices, namely sponge iron (s-Fe^0^), sponge iron + biochar (s-Fe^0^/BC) and sponge iron + biochar + manganese sand (s-Fe^0^/BC/MS), were studied. The morphology and composition changes of sponge iron were analyzed by means of characterization, and the microbial population changes in the three groups were analyzed by high-throughput sequencing. Results showed that the nitrate conversion rate in the s-Fe^0^, s-Fe^0^/BC and s-Fe^0^/BC/MS systems reached 99.48%, 99.57% and 99.36%, respectively, with corresponding ammonia nitrogen generation, rates of 3.77%, 9.34% and 11.24% and nitrogen generation rates of 95.71%, 90.23% and 88.12%. Scanning electron microscopy imaging showed that in the s-Fe^0^/BC and s-Fe^0^/BC/MS systems the surface of sponge iron was highly corroded, with granular substances in the corrosion product clusters. X-ray photoelectron spectroscopy analysis found that the relative contents of Fe_2_O_3_ in the surface oxides of sponge iron after microbial coupling were 38.02% and 71.27% in the s-Fe^0^/BC and s-Fe^0^/BC/MS systems, while the relative Fe_3_O_4_ contents were 61.98% and 28.72%, respectively. Microbial high-throughput sequencing analysis revealed that the Chao and Ace index values in the s-Fe^0^ system were 871.89 and 880.78, while in the s-Fe^0^/BC system they were 1012.05 and 1017.29, and in the s-Fe^0^/BC/MS system were 1241.09 and 1198.29, respectively. The relative proportion of *Thauera* in the s-Fe^0^, s-Fe^0^/BC, and s-Fe^0^/BC/MS systems was 16.76%,14.25% and 10.01%, while the proportion of *Acetoanaerobium* was 15.36%, 13.27% and 11.11%, and the proportion of *Chloroflexi* was 0%, 1.11% and 2.18%, respectively. Furthermore, FAPROTAX function annotation found that the expression levels of chemoheterotrophs in the s-Fe^0^, s-Fe^0^/BC and s-Fe^0^/BC/MS systems were 43 316 OTU, 37 289 OTU and 34 205 OTU, while nitrate respiration expression levels were 16 230 OTU, 15 483 OTU and 9149 OTU, with nitrogen respiration expression levels of 16 328 OTU, 15 493 OTU and 9154 OTU, respectively. These findings suggest that nitrate is converted into nitrogen gas and ammonia nitrogen through the actions of the coupled system of sponge iron/biochar/manganese sand and microorganisms. The catalytic effect of MnO_2_ promotes the conversion of Fe^2+^ to Fe^3+^, generating more electrons, allowing denitrifying bacteria to reduce more nitrate nitrogen, effectively coupling the manganese-catalyzed ICME reaction and microbial denitrification. The micro-electrolysis system and the addition of manganese sand enhanced biodiversity within the s-Fe^0^/BC/MS system. The heterotrophic bacteria *Thauera* and *Acetoanaerobium* were the dominant microorganisms in all three systems, although the micro-electrolysis system with added manganese sand significantly reduced the proportion of facultative bacteria *Thauera* and *Acetoanaerobium* and promoted the growth of autotrophic *Chloroflexi* bacteria. The ecological functions of the three systems were mainly nitrate respiration and nitrogen respiration. By comparing the expression levels of nitrate respiration and nitrogen respiration in s-Fe^0^/BC and s-Fe^0^/BC/MS systems, it can be seen that the addition of manganese sand reduced microbial activity.

## Abstract 2

In a given sample in order to reduce nitrate nitrogen pollution in water, nitrate nitrogen removal methods based on iron–carbon micro-electrolysis have become a key research focus.

## Graphical abstract

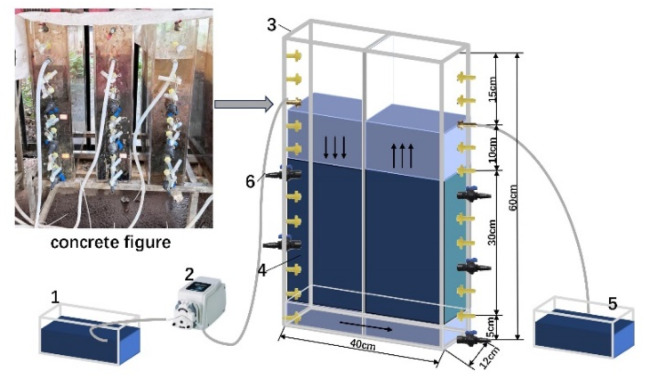

## 1. Introduction

With economic development and the acceleration of industrial urbanization, water resource shortages and the safety of aquatic environments have become important factors limiting social and economic development. Nitrate nitrogen pollution in water is a main factor driving eutrophication, which has both direct and indirect effects on human and environmental health.^1^ Nitrate-nitrogen pertains to the nitrogen component of the overall nitrate present in a given sample therefore, it is essential that effective methods are developed for the reduction of nitrate nitrogen pollution. At present, the treatment methods used to remove nitrate nitrogen from water can be classified as physical, chemical or biological methods. Physical methods use separation technologies such as adsorption, reverse osmosis, electrodialysis and ion exchange to remove nitrate nitrogen, although they cannot change the molecular structure of nitrate nitrogen. Chemical methods use reductants to reduce nitrate nitrogen to NH_4_^+^-N or N_2_,^2^ although by-products continue to be produced, such as NO, NO_2_, NH_2_OH and NH_2_NH_2_. Biological methods convert nitrate nitrogen into nitrogen through microbial heterotrophic denitrification and autotrophic denitrification, in which heterotrophic denitrification requires an organic carbon source, while autotrophic denitrification has a long microbial culture period and low conversion efficiency. At present, ICME technologies are widely used for the treatment of wastewater containing heavy metals, nitrate nitrogen, radionuclides and refractory organic substances, due to its high efficiency, low operational costs and simple operating procedure.^3^ In ICME systems, the iron atom at the negative electrode loses electrons and becomes Fe^2+^, while the electron at the positive electrode H^+^ becomes [H], with both Fe^2+^ and [H] having strong reducibility, providing electrons for nitrate nitrogen reduction and improving the nitrate nitrogen conversion rate.^4^ Previous research has investigated the use of ICME systems without microbial coupling. Jinghuan Luo *et al.*^5^ first used ICME to enhance the reduction of nitrate nitrogen in aqueous solution, achieving a nitrate nitrogen reduction rate of up to 73%, although due to the easy passivation of the iron electrode surface, the nitrogen removal efficiency of the system rapidly decreased. Xuejun Guo *et al.*^6^ reduced nitrate nitrogen by constructing a zero-valent iron/zeolite/oxidant (O_2_, KMnO_4_, H_2_O_2_, CrO_4_^2^) system, which significantly enhanced the reduction of nitrate nitrogen by zero-valent iron and successfully overcame the problem of impedance by the zero-valent iron passivation layer. Because manganese dioxide (MnO_2_), the main component of manganese sand, has a catalytic effect, Guihua Dong *et al.*^7^ found that MnO_2_ can delay the process of passivation of the zero-valent iron surface. Yongqing Sun *et al.*^8^ introduced manganese sand into the ICME system, finding that the addition of manganese sand could strengthen ICME and delay the surface passivation of sponge iron. The surface products were mainly composed of Fe_2_O_3_ and Fe_3_O_4_, with the conversion rate of NO_3_-N reaching 80.3% and the total nitrogen removal rate reaching 58%. Previous research has also focused on ICME systems coupled with microorganisms. For example, Lixia Jia *et al.*^9^ added iron filings and biochar to a constructed wetland system, achieving a nitrate nitrogen reduction rate of 87% due to the ICME process. Jing Tian *et al.*^10^ constructed a two-layer biofiltration system containing biochar in the upper layer and zero-valent iron in the lower layer, finding that the average removal rate of nitrate nitrogen reached 95.7%, which delayed the passivation of zero-valent iron and accelerated the generation of electron donors (H_2_ and Fe^2+^), allowing more NO_3_^−^ to be reduced. Liping Huang *et al.*^11^ combined ICME with aerobic denitrifying bacteria for the treatment of black odor wastewater, finding that ICME could provide electrons for microbial denitrification, reaching a total nitrogen removal rate of 81.49%. Mengyao Hu *et al.*^12^ carried out micro-electrolysis and biological enhanced nitrogen removal treatment under low C/N ratio conditions (C/N = 1.33) and found that the removal rates of total nitrogen and COD were >97% under the conditions of a pH 7 solution and iron–carbon dosage of 10%. Shuai Peng *et al.*^13^ constructed an up flow ICME reactor with microbial coupling and found that the average removal rate of total nitrogen increased from 31.4% to 90.5% after coupling, with XPS analysis showing that microbial coupling accelerated the transformation of Fe^2+^ and Fe_3_O_4_ on the sponge iron surface to FeO(OH), delaying the passivation of sponge iron. However, the mechanism of denitrification in the coupled ICME and microbial system remains unclear.

To date, no studies have reported the addition of manganese sand to an ICME system with microbial coupling for the removal of nitrate nitrogen. In view of this, three continuous flow reactor device groups were designed, containing s-Fe^0^, s-Fe^0^/BC and s-Fe^0^/BC/MS as filler materials. In order to delay the passivation process of the zero-valent iron surface, manganese sand was added to the ICME and microorganisms were added in order to obtain a higher nitrate nitrogen conversion rate. The aim of this study was to compare and monitor the transformation of nitrate nitrogen, as well as the changes in filler composition and microbial population in all three reactor groups, before and after microbial coupling. Furthermore, the transformation path of nitrate nitrogen was analyzed to further reveal the mechanism of nitrogen removal using the mixed filler material coupled with microorganisms, providing a comprehensive understanding of the process of nitrate nitrogen removal from water.

## 2. Materials and methods

### 2.1 Materials

#### 2.1.1 Test apparatus

**Fig. 1:**
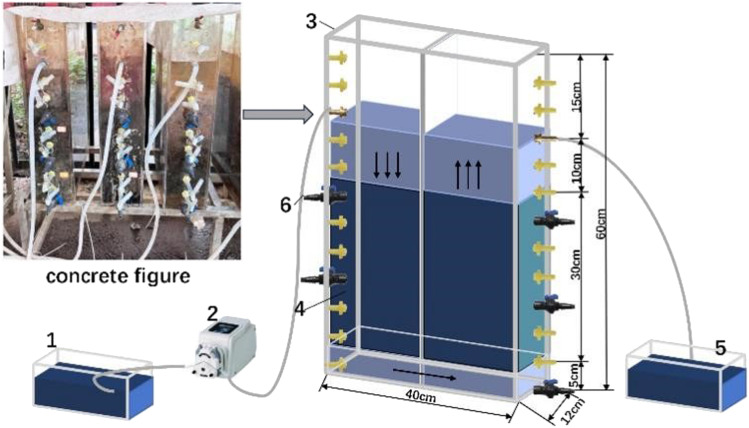
Schematic diagram of the test system setup: (1) storage tank; (2) peristaltic pump; (3) reactor; (4) filler material; (5) outlet tank; (6) sampling valve.

The experimental devices were constructed from 8 mm thick plexiglass, with length × width × height dimensions of 40 cm × 12 cm × 60 cm and a maximum reactor volume of 28 L. Water was divided evenly between the left and right compartments by a partition board, with the base of the system connected to a water collection and distribution space with a height of 5 cm to ensure uniform water distribution. The three reactor groups containing s-Fe^0^, s-Fe^0^/BC, s-Fe^0^/BC/MS filler materials were all filled to achieve equal volumes of 14.4 L and thicknesses of 30 cm. A continuous flow system was used, in which the solution was pumped into the upper surface of one side of the device *via* a peristaltic pump at a flow rate of 7 ml min^−1^. The downward flow entered the other side of the system through the water collection and distribution space, was then converted into upward flow, overflowing after passing through the packing and finally, was discharged through the drain. A schematic diagram of the experimental device is shown in Fig. 1.

#### 2.1.2 Experimental materials

**Fig. 2:**
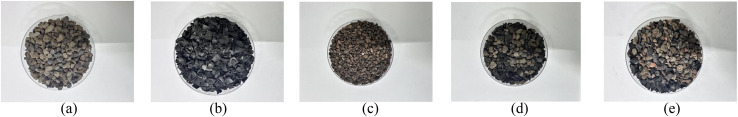
Experimental materials: (a) s-Fe^0^; (b) BC; (c) MS; (d) s-Fe^0^/BC (3 : 1); (e) s-Fe^0^/BC/MS (6 : 2 : 1).

All reagents used were of high purity grade and were purchased from Komio Chemical Reagent Co. Ltd. (Tianjin, China). All aqueous solutions were prepared using deionized water with the electrical conductivity of 15–18 Ω cm. Sponge iron was purchased from Henan Weikotli Environmental Protection Technology Co. Ltd. (Henan, China), which had a diameter of 3–5 mm and an active iron content of >98%. Biochar formed from the high temperature activation and carbonization treatment of high-quality coconut shell activated carbon, had a particle size of 4–8 mesh and was purchased from Henan Zhengjie Environmental Protection Technology Co. Ltd. (Henan, China). Manganese sand (55%-MnO_2_, 25%-SiO_2_, 20%-Fe) was purchased from Henan Zhengjie Environmental Protection Technology Co. Ltd. (Henan, China), with a particle size of 1–2 mm. All materials were washed three times with deionized water and then dried for later use (Fig. 2).

### 2.2 Methods

#### 2.2.1 Removal of nitrate nitrogen before and after coupling of sponge iron/biochar/manganese sand with microorganisms

##### 2.2.1.1 Physicochemical experiments

According to preliminary static tests, it was found that the reaction equilibrium could be reached in 6 h, so in all experiments the Hydraulic Retention Time (HRT) was controlled to 6 h. The experiment was run for 6 days at a temperature of 24.2 °C, without aeration. Various parameters and indices were measured to study the transformation law of nitrate nitrogen in the three reactor groups. The concentration of nitrate nitrogen in the influent was 58.2–60.3 mg L^−1^, the pH was 7.1–7.2 and Dissolved Oxygen (DO) concentration was 8.62–8.8 mg L^−1^.

##### 2.2.1.2 Biological coupling operational experiments

After the three reactors were inoculated and stable operation was achieved, experimental analysis was carried out. The transformation law of nitrate nitrogen in different systems was studied under the condition of microbial coupling. The activated sludge used for inoculation was supplied from the secondary sedimentation tank of a sewage treatment plant in Xi'an University of Science and Technology (China). After immersion and standing for 24 h, the period of continuous operation was initiated, with an operating temperature of 24.2 °C, an HRT of 6 h and without aeration. The continuous operation period lasted for 37 days, proceeding through two stages of start-up and stable operation, in which the start-up stage lasted for 15 days and the stable operation stage lasted for 22 days. Synthetic water samples were prepared to contain an influent nitrate nitrogen concentration of 80 mg L^−1^, with a carbon to nitrogen mass ratio of 3 : 1 and a pH of 7.0 maintained using NaHCO_3_. Table 1 shows the specific influent composition and the degree of fluctuation in the actual test data.

**Table 1:** Table of quick-start water distribution components

Wastewater components	Concentration (mg L^−1^)
NO_3_^−^-N	80.0
COD	240.0
NaHCO_3_	2000.0
Constant element	KH_2_PO_4_ (17.0)
	MgCl_2_ (60.0)
	CaCl_2_ (33.2)
Trace element	ZnSO_4_·7H_2_O (0.1109)
	Na_2_MoO_4_·2H_2_O (0.110)
	CoCl_2_·6H_2_O (0.120)
	MnSO_4_·H_2_O (0.117)
	NiCl_2_·6H_2_O (0.104)
	Na_2_HPO_4_ (0.570)
	FeCl_3_·6H_2_O (0.124)
Rhamnolipid	30.0 (mixture of mono- and di-rhamnolipids in a weight ratio of 1 : 1.5) (continuous dosing for 5 days at the start-up stage)

#### 2.2.2 Microbial high-throughput sequencing experiments

In order to explore the changes in microbial community composition before and after microbial coupling in the three reactor systems, the influence of adding manganese sand on microbial community formation and the influence of microbial community changes in different fillers on the nitrogen removal process were explored. After the stable operation period, biofilm samples A1 (#1), A2 (#2) and A3 (#3) were collected from the filler material 10 cm below the upper surface of the water inlet side of reactors #1, #2 and #3. Biological samples were sequenced using a high-throughput sequencing platform, with sequence analysis and species annotation carried out according to a bioinformatics analysis method, in order to comprehensively analyze the microbial species, quantity, diversity and community composition in each system. The functional population groups were obtained using FAPROTAX functional annotation.

### 2.3 Characterization and analysis

#### 2.3.1 Analysis methods

The standard method of APHA (1998) was used to analyze nitrate nitrogen, nitrite nitrogen, ammonia nitrogen and COD in the solution. The total nitrogen (TN) content was calculated according to the sum of NH_4_^+^-N, NO_3_-N, NO_2_-N and N_2_. SEM (Quanta 250 FEG, FEI, USA) was used to investigate the changes in surface morphology of sponge iron after microbial coupling in the ICME system. XPS (ESCALAB Xi+, Thermo Fisher Scientific, USA) analysis was used to determine differences in iron oxide types and relative content on the sponge iron surface after the filler was coupled with microorganisms.

In the experiment, effluent nitrogen, NO and N_2_O concentrations were determined. N_2_O was not detectable by gas chromatography (CG-2014, Shimadzu, Japan) and it has previously been reported that almost no NO_*x*_ or N_2_O are produced during the reduction of nitrate nitrogen.^14,15^ Therefore, by default all gaseous nitrogen-containing substances in this study were N_2_. The corresponding selectivity calculation formulas for ammonia nitrogen, nitrite nitrogen and nitrogen are described by eqn (2-1)–(2-3), respectively:2-1
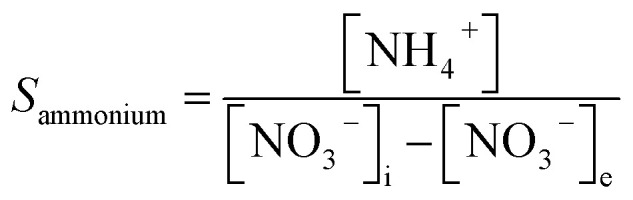
2-2
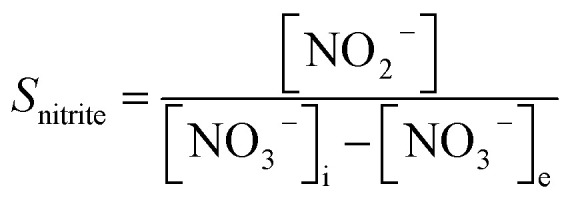
2-3
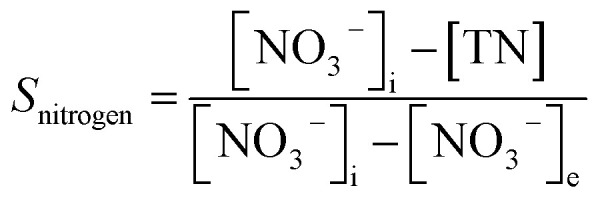
where, *S*_ammonium_, *S*_nitrite_ and *S*_nitrogen_ indicate the selectivity of ammonia nitrogen, nitrite and nitrogen; [NH_4_^+^], [NO_2_^−^] and [TN] are the concentrations of residual ammonia-nitrogen, nitrite and total nitrogen in solution, respectively; [NO_3_^−^]_i_ and [NO_3_^−^]_e_ are the initial and residual nitrate concentrations in solution.

## 3. Results and discussion

### 3.1 Transformation and removal of N by physical and chemical pathways in different filler materials

**Fig. 3:**
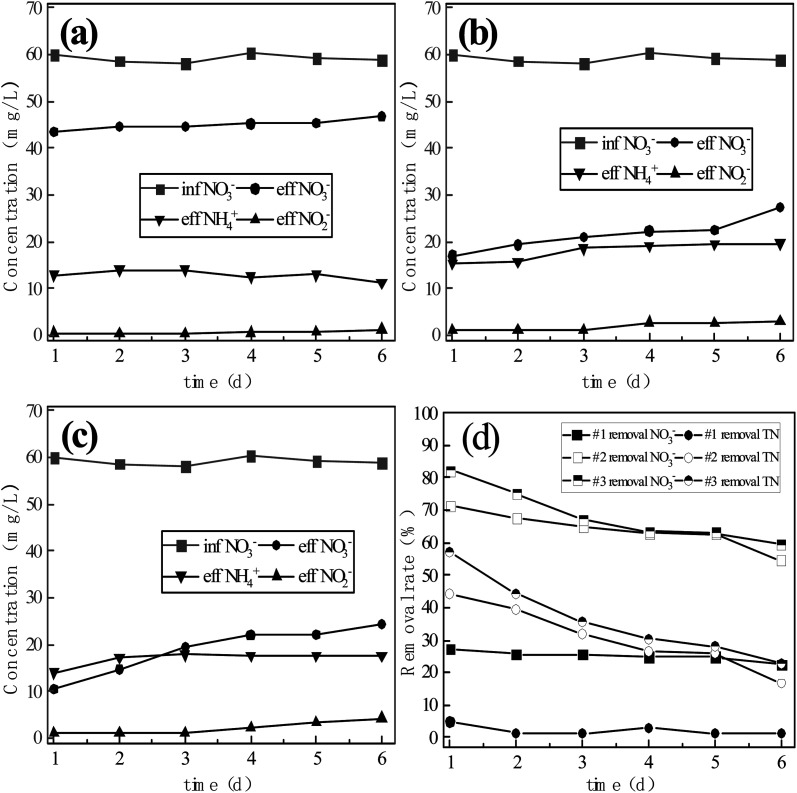
(a) Nitrogen transformation by chemical denitrification in reactor #1; (b) nitrogen transformation by chemical denitrification in reactor #2; (c) nitrogen transformation by chemical denitrification in reactor #3; (d) nitrate nitrogen transformation and total nitrogen removal by chemical denitrification.

The nitrogen removal and conversion rates measured from physicochemical interactions in the three reactor systems without microbial inoculation are shown in Fig. 3. The continuous flow reaction was carried out for 6 days with an influent nitrate nitrogen concentration of 60 mg L^−1^. With ongoing reaction time, the concentration of nitrite nitrogen in the effluent tended to increase and accumulate. The maximum concentrations of nitrite nitrogen in the effluent of reactors #1, #2 and #3 were 1.35 mg L^−1^, 3.10 mg L^−1^ and 4.31 mg L^−1^. The average concentrations of ammonia nitrogen in the effluent of reactors #1, #2 and #3 reactors were 13.02 mg L^−1^, 18.05 mg L^−1^ and 17.11 mg L^−1^, while the maximum concentrations were 14.11 mg L^−1^, 19.72 mg L^−1^ and 17.91 mg L^−1^, showing that the effluent ammonia nitrogen concentrations were relatively stable. From the above data, it can be seen that the effluent ammonia nitrogen concentrations of reactors #2 and #3 were very similar, while the effluent ammonia nitrogen concentration from reactor #1 was significantly lower than those of reactors #2 and #3. It may be speculated that nitrate nitrogen was more easily reduced in the ICME system because micro-electrolysis delays the process of filler surface passivation, improving the retention of active sites on the filler surface. By comparing the concentrations of ammonia nitrogen in the effluent of reactors #2 and #3, it can be seen that the addition of manganese sand had no obvious effect on the formation of ammonia nitrogen. It can be inferred that Fe in the ICME system is more likely to lose electrons to provide H^+^ ions and generate [H], which promotes the reduction of nitrate nitrogen and the dissolution of surface corrosion products, effectively delaying the passivation of the filler surface and maintaining a high reduction activity. Tasuma Suzuki *et al.*^16^ previously reported that accelerating iron corrosion can increase the specific surface area of sponge iron, thus increasing the degree of nitrate nitrogen reduction, while Fei Xie *et al.*^17^ found that ICME produced a high abundance of soluble Fe^2+^, which could promote the reduction of nitrate nitrogen. These previous studies reflect that the ICME system can accelerate iron corrosion, delaying filler surface passivation and strengthening nitrate nitrogen reduction.

In terms of nitrate removal, with ongoing reaction time the concentration of nitrate nitrogen in the effluent of reactor #1 increased from 43.51 mg L^−1^ to 46.77 mg L^−1^, corresponding to a decrease in transformation rate from 27.39% to 22.36%. The concentration of nitrate nitrogen in the effluent of reactor #2 increased from 17.09 mg L^−1^ to 27.41 mg L^−1^, corresponding to a reduction in the conversion rate from 71.48% to 54.51%. The concentration of nitrate nitrogen in the effluent of reactor #3 increased from 10.56 mg L^−1^ to 24.46 mg L^−1^, corresponding to a decrease in the conversion rate from 82.37% to 59.40%. Therefore, system #3 achieved the best nitrate nitrogen conversion effect. With ongoing reaction time, the conversion efficiency of nitrate nitrogen in the three reactor groups decreased by varying degrees, with the greatest effect observed in systems #2 and #3. However, after the same reaction duration, the conversion rate of nitrate nitrogen was consistently higher in system #3 than in system #2, while #2 was better than #1. It may be speculated that with the extended reaction duration the surface of sponge iron is gradually passivated and the active sites required for nitrate nitrogen reduction decrease. The addition of biochar can accelerate the corrosion process and delay the passivation of sponge iron, while the addition of manganese sand further delays the passivation process. This is consistent with the findings of previously reported research in this area.^8^ In terms of total nitrogen removal, system #1 achieved a reduction from 4.7% to 1.2%, indicating that the nitrogen removal capacity of pure sponge iron through physical and chemical mechanisms was very limited. The total nitrogen removal rates of systems #2 and #3 also decreased with ongoing reaction duration, with total nitrogen removal rates decreasing from 44.1% to 16.6% and 56.9% to 27.2%, respectively, after 6 days of operation. The addition of biological activated carbon and manganese sand improved the nitrogen removal efficiency of the system, as reducing substances (Fe^2+^, [H]) are produced by ICME, while the MnO_2_ in manganese sand accelerated the transformation of zero-valent iron into Fe^2+^ and accelerated the corrosion of sponge iron. Even after passivation, the total nitrogen removal performance of the ICME system containing manganese sand was still better than that of the ICME-only system.

Therefore, the addition of BC and MS to the s-Fe^0^ system significantly improved nitrate nitrogen reduction and total nitrogen removal. Sponge iron became passivated in all three reactor groups with ongoing reaction time and the reducing active sites for nitrate nitrogen decreased, resulting in a decline in nitrate nitrogen conversion rate and total nitrogen removal rate to varying degrees in each system.

### 3.2 Conversion and removal of N by coupling of different fillers and microorganisms

**Fig. 4:**
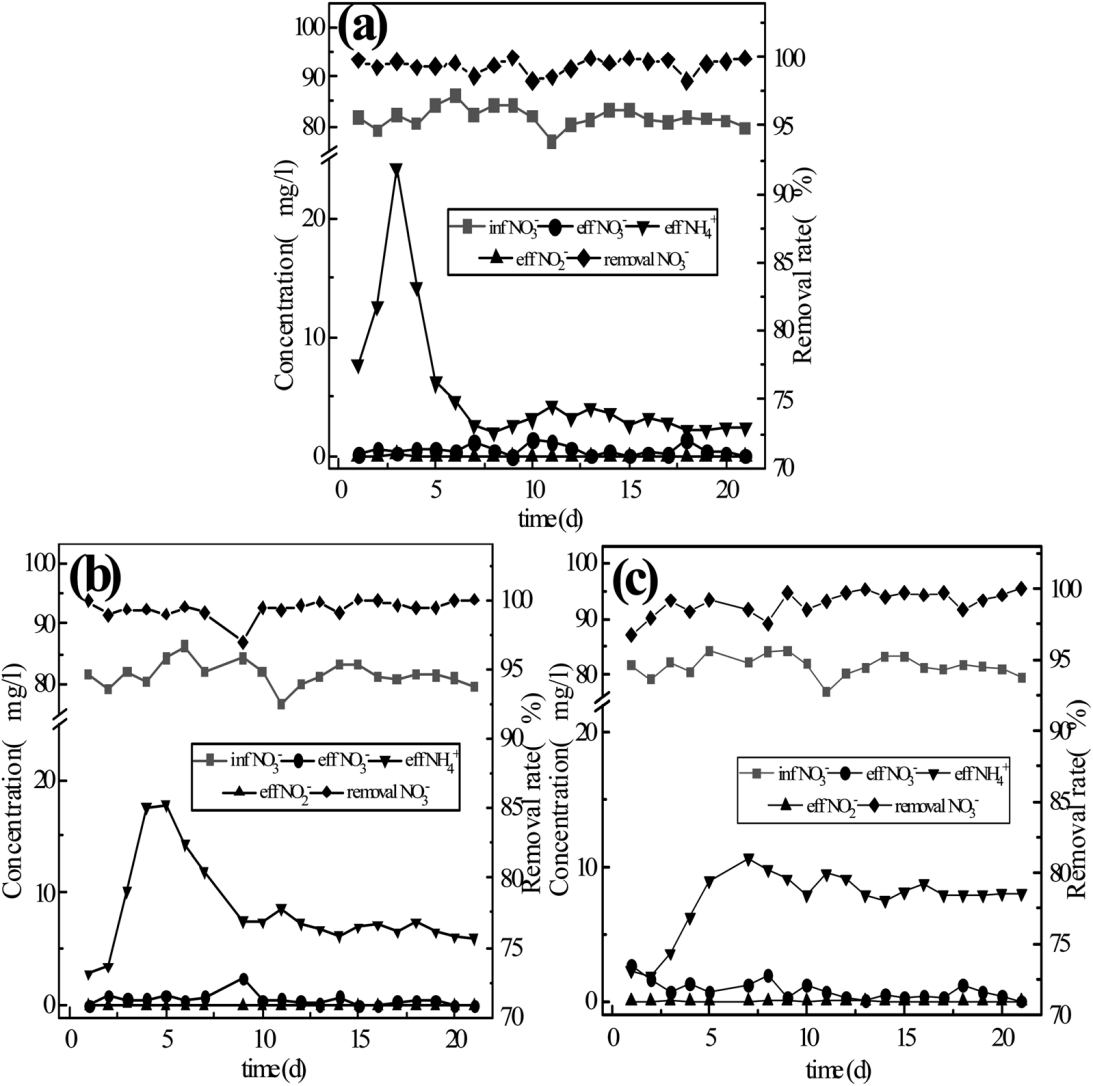
Conversion of nitrate by the three-component system with microbial coupling: (a) reactor #1: s-Fe^0^; (b) reactor #2: s-Fe^0^/BC; (c) reactor #3: s-Fe^0^/BC/MS.

In order to explore the mechanism of nitrogen transformation and removal by the coupling of different fillers and microorganisms, activated sludge was inoculated into three sets of reactors that had been running for 6 days previously, with continuous operation treating a synthetic water supply for 37 days. Chong Peng *et al.*^14^ previously found that suitable concentration of rhamnolipids could accelerate the start-up of low C/N ratio reactor systems and shorten the time required for film-formation, so in the initial five days of inoculation, rhamnolipids were added to the synthetic water to shorten the film-forming duration. After operating for 15 days, each reactor reached stability. The monitored results are shown in Fig. 4.

As shown in Fig. 4, the average concentrations of nitrate nitrogen in the effluent of reactor systems #1, #2 and #3 were 0.64 mg L^−1^, 0.45 mg L^−1^ and 0.40 mg L^−1^, respectively, with the average conversion rate of nitrate nitrogen reaching 99.48%, 99.57% and 99.36%, among which the lowest nitrate nitrogen concentration in the effluent of system #2 reached 0.04 mg L^−1^, corresponding to the highest average conversion rate. After microbial coupling, the conversion rate of nitrate nitrogen was very high in all three reactors, with the nitrate nitrogen conversion rate being highest in reactor #2. On the 6th day of operation, the nitrate nitrogen conversion rates established under continuous flow in reactors #1, #2 and #3 were 22.36%, 54.51% and 59.40%, respectively. Microbial coupling was found to significantly improve the conversion efficiency of nitrate nitrogen, increasing conversion rates by 77.12%, 45.06% and 39.96%. Under low carbon nitrogen ratio (C/N < 4) conditions,^18^ the microbial denitrification effect was low and the total nitrogen removal rate was 48.6% ± 6%, as under these conditions there were insufficient electron donors available in the water, limiting microbial denitrification. The coupling of ICME and microorganism can provide more electron donors for microbial denitrification, due to the corrosion of anode metals,^19–21^ while simultaneously the cathode produced an abundance of [H], which can also provide more electron donors for the denitrification process and achieve the efficient transformation of nitrate nitrogen. Compared with the ammonia nitrogen yield of 21.7%, 30.1% and 28.5% achieved in systems #1, #2 and #3 due to pure physical and chemical action (without microbial coupling), the ammonia nitrogen yield of the system after microbial coupling decreased to 3.77%, 9.34% and 11.24%. Therefore, microbial coupling could greatly reduce the yield of ammonia nitrogen and realize biological denitrification. The micro-electrolysis process causes Fe to release electrons, producing Fe^2+^ and Fe^3+^, which may contribute to electron transfer during denitrification. Xiaoying Zheng^22^ reported that Fe^2+^ and Fe^3+^ produced in the ICME process could be used as an electron shuttle to improve the efficiency of biochemical reactions, while manganese dioxide can catalyze Fe^2+^ to generate Fe^3+^ and release electrons, with both of these processes being beneficial to electron release. Furthermore, N in the system can be used as an electron acceptor, resulting in the potential generation of NH_4_^+^-N. However, during this process, the rate of ammonia nitrogen production decreased significantly, which may be due to biofilm growth reducing active sites on the surface of sponge iron and increasing the mass transfer resistance of the matrix, thus limiting the chemical denitrification of nitrate nitrogen by the filler material.

As can be seen from Fig. 4, once stable operation was achieved, the nitrite nitrogen concentration in the effluent of reactors #1, #2 and #3 was 0 mg L^−1^. Compared with the physical and chemical process without microbial coupling, nitrite nitrogen was completely reduced in the microbial coupled system without any observed accumulation. In the first 5 days after the addition of sludge, the effluent concentration of ammonia nitrogen increased sharply, possibly due to the endogenous respiratory metabolism of inoculated activated sludge. After stable operation was achieved, the effluent concentration of ammonia nitrogen exhibited no obvious fluctuations, with average values of 2.72 mg L^−1^, 6.69 mg L^−1^ and 8.03 mg L^−1^, while the average ammonia nitrogen production rates were 3.77%, 9.34% and 11.24%. Based on the above data, in the previous physical and chemical experiments without microbial coupling, the average effluent ammonia nitrogen concentrations in the three reactor groups were 11.32 mg L^−1^, 19.72 mg L^−1^ and 17.78 mg L^−1^, with ammonia nitrogen production rates on the 6th day being 19.23%, 33.49% and 30.20%. Huan Zhang *et al.*^23^ previously reported that during the process of nitrate reduction by single nanometer zero-valent iron, a large amount of ammonia nitrogen accumulated within the system. In the present study, after the three reactor groups were coupled with microorganisms, the ammonia nitrogen generation rate was significantly reduced, and the ammoniation of nitrate nitrogen was inhibited. It may be speculated that in the microbial coupled system, the active sites on the filler surface required for nitrate nitrogen reduction are further reduced due to the formation of a biofilm. In addition, in the presence of organic carbon sources, denitrifying microorganisms strengthen heterotrophic denitrification. Seok-Younohe *et al.*^15^ reported that when anaerobic microorganisms are present, the products of the reaction between nZVI and NO_3_-N are gaseous nitrogen (NO, NO_2_, N_2_), rather than NH_4_^+^-N or NO_2_^−^-N. Due to the degradation and mass transfer resistance of biofilms formed on the surface of the filler, the amount of nitrate nitrogen that can ultimately reach the surface of the filler is greatly reduced, thus weakening the process of nitrate nitrogen transformation into ammonia nitrogen due to physical and chemical effects, resulting in a reduction in the effluent concentration of ammonia nitrogen. Overall, the results of these experiments show that the addition of BC and MS to the filler material could promote the transformation of nitrate nitrogen into ammonia nitrogen.

**Fig. 5:**
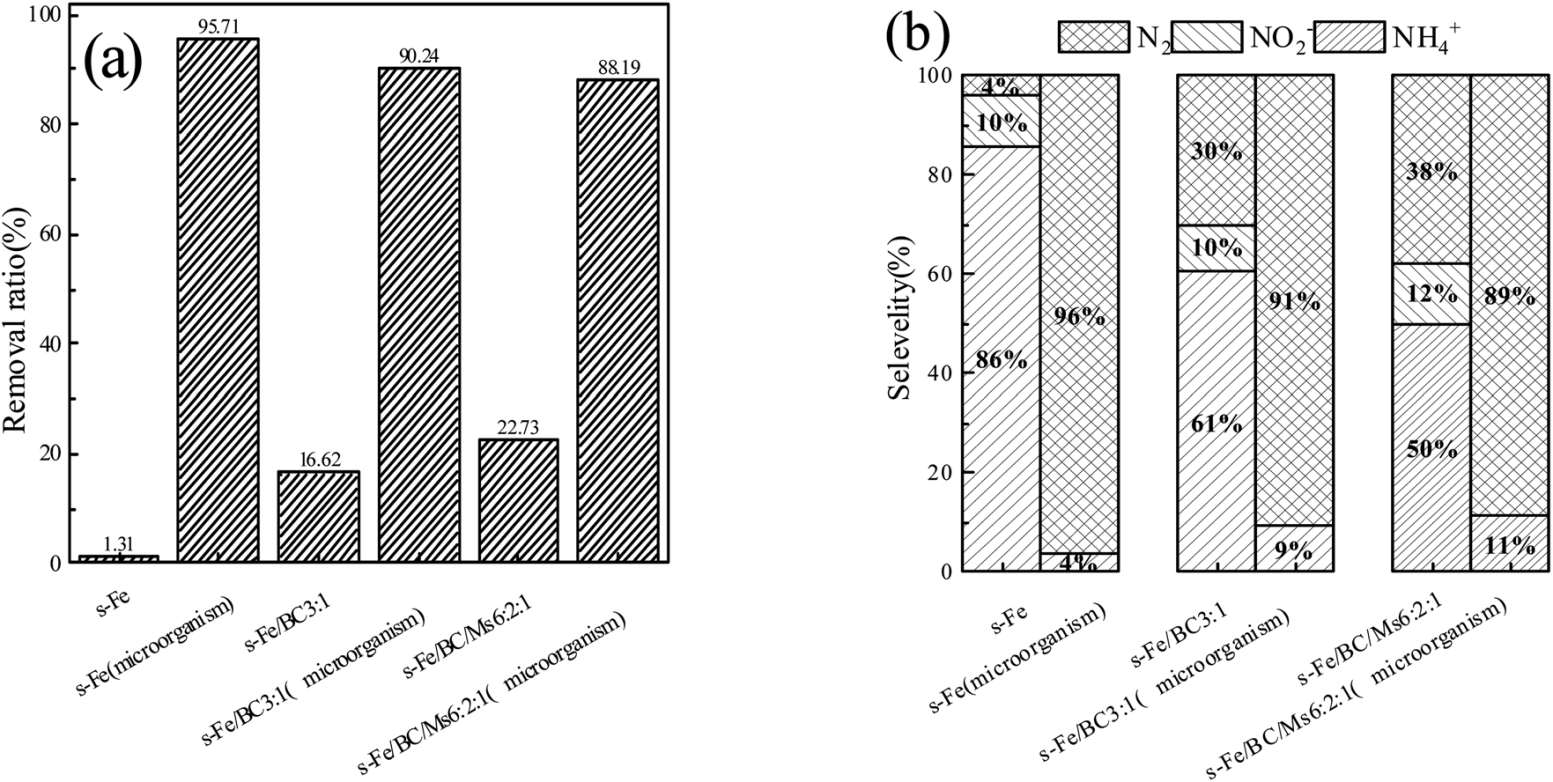
(a) TN removal rate before and after microbial coupling in three reactor groups; (b) proportion of nitrogen-containing products before and after microbial coupling in the three reactor groups.

According to Fig. 5(a), the total nitrogen removal rate before microbial coupling was ranked in the descending order of: #3 (22.73%) > #2 (16.62%) > #1 (1.31%), with the rate of total nitrogen removal by reactor #3 and #2 being much higher than that of reactor #1 and #3 being significantly higher than that of #2. After stable operation was achieved in the microbial coupled systems, the total nitrogen removal rate before microbial coupling was ranked in the descending order of: #1 (95.71%) > #2 (90.24%) > #3 (88.19%). After the introduction of biological denitrification, the rate of total nitrogen removal by reactor #1 was significantly better than by reactors #2 and #3, with reactor #2 having a slightly higher rate than reactor #3. Compared with physical and chemical actions only, microbial coupling significantly improved the rate of total nitrogen removal, achieving an increase in nitrogen removal rates in reactors #1, #2 and #3 by 94.40%, 73.62% and 65.46%, respectively. From the above data, it can be seen that the pure sponge iron system had no significant inhibitory effect on biological denitrification, although biological denitrification was reduced after the addition of activated carbon and a greater reduction was observed after the addition of activated carbon and manganese sand. Sihai Hu *et al.*^24^ found that after activated carbon was introduced into the filler, the ICME effect was formed and the amount of electron donors provided to autotrophic denitrifying microorganisms was increased, resulting in an improved autotrophic denitrification efficiency. However, manganese sand is unfavorable for the growth of *Thauera*. According to the research of Zichun Yan *et al.*,^25^ excessive concentrations of Mn^2+^ inhibit *Thauera*, an important facultative autotrophic denitrifying bacteria. When the organic matter content was sufficient, organic carbon sources are used for heterotrophic denitrification. However, with the increase in Mn^2+^ concentration in the present study, the relative abundance of some heterotrophic denitrifying microbes decreased, which was not conducive to heterotrophic denitrification. Therefore, it may be speculated that different filler materials have different effects on the growth of heterotrophic and autotrophic denitrifying bacteria, leading to obvious differences in denitrification performance of the three systems.

In order to further study the coupled mechanism of nitrate nitrogen reduction by different fillers and microorganisms, the relative proportions of nitrogen-containing products were comprehensively studied in three reactor groups before and after microbial coupling, as shown in Fig. 5(b). On the 6th day of operation without microbial coupling, reactors #1, #2 and #3 were able to effectively transform nitrate nitrogen to ammonia nitrogen (accounting for 85.64%, 60.52% and 50.04% of total nitrogen, respectively), nitrite nitrogen (accounting for 10.28%, 9.53% and 12.14% of total nitrogen, respectively) and nitrogen (accounting for 4.07%, 29.96% and 30.04% of total nitrogen, respectively). This is consistent with the previously reported findings of Ningfan Song and Liang Wu, showing that the reduction product of the ICME system was mainly NH_4_^+^-N.^26,27^ After stable operation was achieved in the microbial coupled systems, the average proportions of ammonia nitrogen converted in reactors #1, #2 and #3 were 3.79%, 9.38% and 11.31%, respectively, with no nitrite nitrogen accumulated, while the average proportions of nitrogen converted were 96.21%, 90.62% and 88.69%, respectively. After microbial coupling, the ammonia nitrogen production rate in the three reactor groups decreased significantly, while the nitrogen production rate increased significantly. By analyzing the data, it was found that nitrate nitrogen ammoniation was the main process involved in chemical denitrification, with ammonia nitrogen being the main product and nitrite nitrogen accumulated to some extent. After microbial coupling, biological denitrification became the dominant process, with nitrate nitrogen mainly converted into nitrogen and nitrite nitrogen not accumulated. This further supports that microbial coupling can inhibit ammoniation and reduce ammonia nitrogen production, with this process probably caused by two reasons: (1) biofilm growth and coverage leads to a reduction in active sites for chemical denitrification on the surface of the filler material; (2) after microbial coupling, the concentration of nitrate nitrogen in the solution decreased after biological denitrification and simultaneously, the biofilm on the surface of the filler material increased the mass transfer resistance of nitrate nitrogen to the filler surface, decreasing the nitrate nitrogen concentration able to pass through the biofilm and reach the filler surface, reducing chemical denitrification.

### 3.3 Characterization of the sponge iron surface in the iron–carbon micro-electrolysis system

**Fig. 6:**
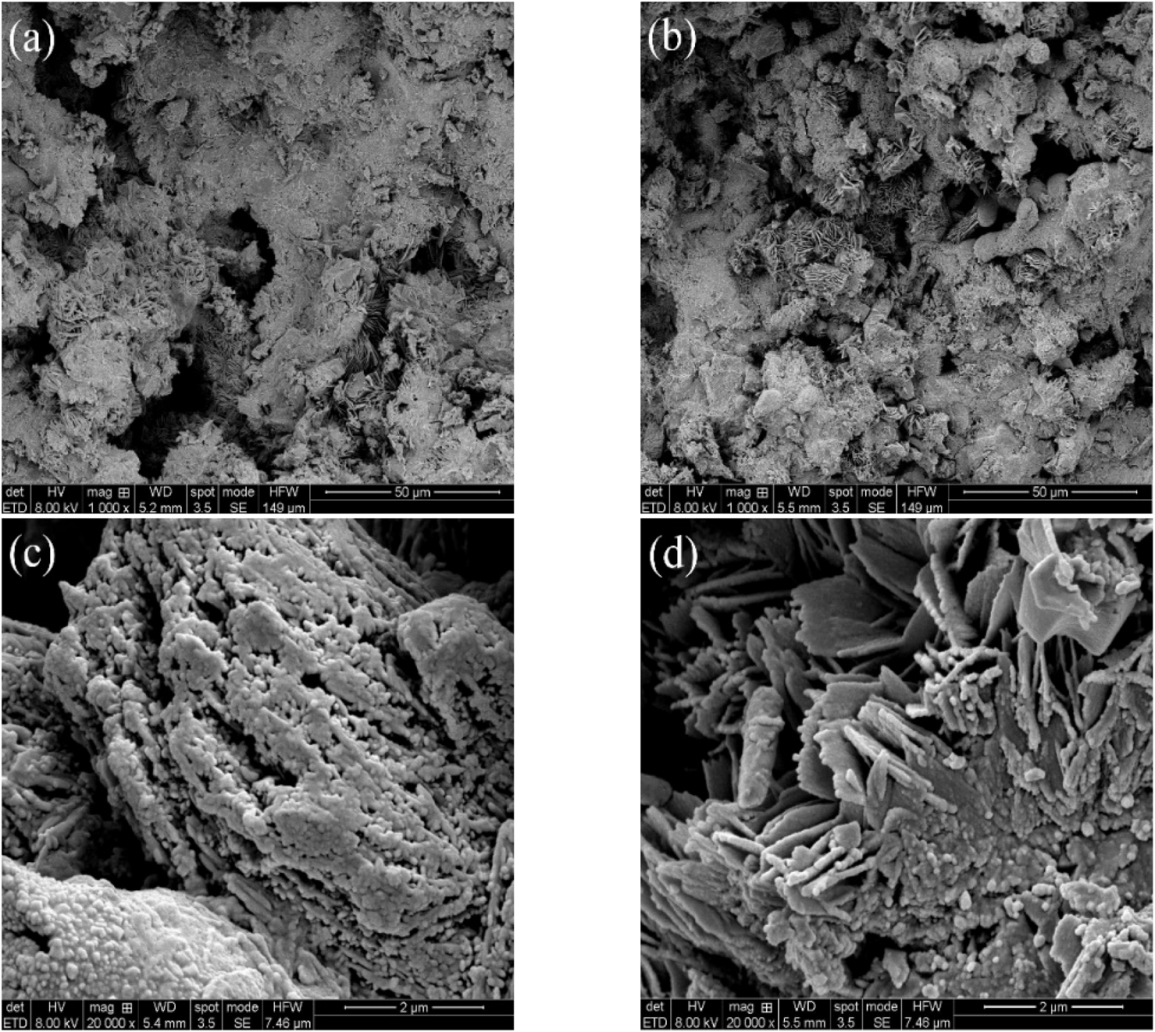
SEM images of the sponge iron surface in (a) the s-Fe^0^/BC microbial coupled system (×1000 magnification); (b) the s-Fe^0^/BC/MS microbial coupled system (×1000 magnification); (c) the s-Fe^0^/BC microbial coupled system (×20 000 magnification); (d) the s-Fe^0^/BC/MS microbial coupled system (×20 000 magnification).

In order to investigate the surface morphology changes of sponge iron after microbial coupling in the ICME system, the sponge iron in systems #2 and #3 were characterized by SEM, as shown in Fig. 6(a)–(d). As shown in Fig. 6(a), the surface of sponge iron in the s-Fe^0^/BC and s-Fe^0^/BC/MS coupled microbial systems was observed by SEM with 1000 times magnification, showing that no corrosion could be observed on the surface of sponge iron in the s-Fe^0^/BC system, while the degree of sponge iron corrosion in the s-Fe^0^/BC/MS system was enhanced. As shown in Fig. 6(c) and (d), when the magnification was adjusted to 20 000 times some granular substances were found to be produced on the surface of sponge iron in both systems, with the surface structure of sponge iron in the s-Fe^0^/BC/MS system visibly looser, with a larger specific surface area. Compared with the SEM images of sponge iron in the s-Fe^0^, s-Fe^0^/BC and s-Fe^0^/BC/MS fillers reported by Yongqing Sun *et al.*,^8^ it was found that the sponge iron surface in both the s-Fe^0^/BC and s-Fe^0^/BC/MS systems were more strongly corroded after microbial coupling, with the surfaces of corrosion product clusters covered, potentially by dehydrated biofilms, as indicated by SEM imaging. From Fig. 6, it can be seen that both the s-Fe^0^/BC and s-Fe^0^/BC/MS systems provided a good environment for the growth of microorganisms, which is beneficial for biofilm development. However, the extent of coverage was significantly reduced in the s-Fe^0^/BC/MS system, which may be due to the toxic effect of manganese sand on microorganisms. As can be seen from the figure, when coupled with microorganisms, it can be observed that a large number of microorganisms adhere to the surface of sponge iron in s-Fe^0^/BC and s-Fe^0^/BC/MS systems, and these microorganisms may form a biofilm covering the original surface of sponge iron. In addition, the growth and metabolic activities of microorganisms may lead to more holes and irregular structures on the surface of sponge iron, which increases the surface area of sponge iron, improves the contact area with wastewater, and provides a good living space for microorganisms, which is conducive to the growth of biofilm. At the same time, these microorganisms may also participate in the process of electron transfer and material exchange, thus affecting the transformation of nitrate and improving the transformation effect of nitrate nitrogen. However, the covered matter in s-Fe^0^/BC/MS system is obviously reduced, which may be due to manganese sand. Yibo Wang *et al.*^28^ used the ICME method to couple nitrifying bacteria and denitrifying bacteria, finding that some granular bacteria appeared in SEM images and that the system easily formed a highly active biofilm which maintained a high biomass within the reactor, which is consistent with the findings of the present study. We also found that there are more layered sponges in Fig. 6(d) compared with Fig. 6(c), which may be due to the fact that manganese sand promotes iron–carbon micro-electrolysis and accelerates the corrosion of sponge iron surface, resulting in more layered sponges on sponge iron surface. Manganese sand plays a catalytic and adsorption role in the system, which can promote the degradation of organic matter and the reduction of nitrate in wastewater. The porous structure and good adsorption of sponge iron can provide rich attachment sites for microorganisms, leading to microorganisms in certain areas. This layered structure is beneficial to the full contact and interaction between microorganisms and sponge iron, and improves the efficiency of electron transfer and material exchange.

**Fig. 7:**
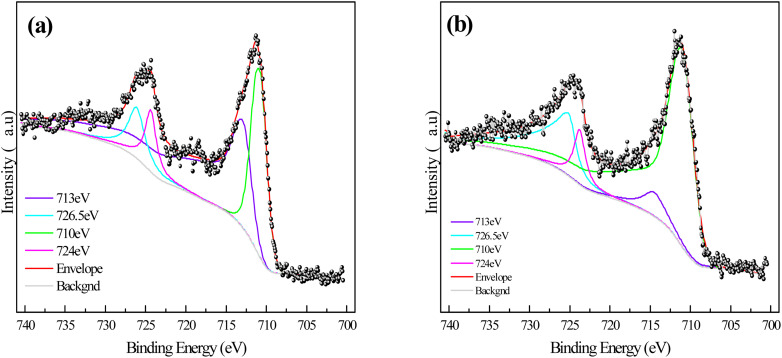
XPS images of the sponge iron surface in the combined filler systems after microbial coupling: (a) s-Fe^0^/BC; (b) s-Fe^0^/BC/MS.

In order to explore the types and amounts of corrosion products on sponge iron surface after microbial coupling of the s-Fe^0^/BC and s-Fe^0^/BC/MS systems, the corrosion products on the sponge iron surface were collected and analyzed by XPS. Fig. 7(a) and (b) show the XPS spectra of Fe 2p on the sponge iron surface use in the s-Fe^0^/BC and s-Fe^0^/BC/MS microbial coupled systems. The observed peaks at 713 eV and 726.5 eV represent Fe_3_O_4_, while the peaks at 710 eV and 724 eV represent Fe_2_O_3_. The relative content of iron elements with different valence states in the surface oxides of sponge iron in the two systems are shown in Table 2. By comparing the relative contents of Fe_2_O_3_ and Fe_3_O_4_ in the s-Fe^0^/BC and s-Fe^0^/BC/MS systems, it was found that Fe_2_O_3_ increased from 38.02% to 71.27%, while Fe_3_O_4_ decreased from 61.98% to 28.72%. Furthermore, MnO_2_ in manganese sand served as a catalyst, promoting Fe^2+^ to Fe^3+^ and resulting in a decrease in Fe_3_O_4_ content and an increase in Fe_2_O_3_ content. Yongqing Sun *et al.*^8^ studied the transformation of nitrate nitrogen by s-Fe^0^/BC and s-Fe^0^/BC/MS under pure physical and chemical action and found that the content of Fe_2_O_3_ in the surface corrosion products of sponge iron in the s-Fe^0^/BC and s-Fe^0^/BC/MS systems increased from 34% to 55.7%, while Fe_3_O_4_ decreased from 66% to 44.3. Compared the experimental results with in the present study, these findings show that microbial coupling can promote the corrosion and oxidation of iron in s-Fe^0^/BC and s-Fe^0^/BC/MS systems. As a result, the relative content of Fe_2_O_3_ in corrosion products increased, while the relative content of Fe_3_O_4_ decreased. It may be speculated that Fe^2+^ and Fe^3+^ produced during the ICME process may form electron shuttles, ultimately improving the efficiency of biochemical reactions. This is in agreement with a previous report by Xiaoying Zheng,^22^ showing that Fe^2+^ and Fe^3+^ produced during ICME can be used as electron shuttles. Fe^3+^ in corrosion product Fe_2_O_3_ is a strong oxidant, which has the ability to accept electrons and be reduced. Fe^3+^ can be used as an electron acceptor to obtain electrons from other substances (such as organic substances or microorganisms) during the reduction of nitrate nitrogen, and gradually reduce nitrate nitrogen to nitrite, ammonia nitrogen or other forms of nitrogen.

**Table 2:** Relative content of different forms of Fe species on the sponge iron surface after use in the s-Fe^0^/BC and s-Fe^0^/BC/MS systems with microbial coupling

Fe substance	s-Fe^0^/BC	s-Fe^0^/BC/MS
Fe-Fe_2_O_3_	38.02%	71.27%
Fe-Fe_3_O_4_	61.98%	28.72%

To sum up, SEM results show that the surface of sponge iron in s-Fe^0^/BC system is obviously corroded after coupling microorganisms, and the surface corrosion degree of sponge iron in s-Fe^0^/BC/MS system is enhanced. The increased corrosion of sponge iron can increase the active sites on the surface, thus improving the transformation efficiency of nitrate nitrogen. At the same time, both systems provide good living space for microorganisms, making the microorganisms on the surface grow better, improving their biological denitrification, and thus improving the nitrogen removal efficiency. XPS analysis showed that coupled microorganisms enhanced the corrosion and oxidation of sponge iron in s-Fe^0^/BC and s-Fe^0^/BC/MS systems, resulting in an increase in the relative content of Fe_2_O_3_ and a decrease in the content of Fe_3_O_4_. Fe^2+^ and Fe^3+^ produced during ICME acted as electron shuttles, which accelerated the electron transfer and improved the efficiency of biochemical reaction. As a strong oxidant, Fe^3+^ in Fe_2_O_3_ can receive electrons and be reduced, thus participating in the reduction of nitrate nitrogen and transforming it into nitrite, ammonia nitrogen and other forms.

### 3.4 Analysis of the biological population on the surface of different filler materials

#### 3.4.1 Microbial community richness and diversity analysis

Analysis of nitrate nitrogen reduction products from different stages, showed that the filler material coupled with microorganisms had a significant impact on the nitrate nitrogen reduction process. By establishing the richness and diversity of microbial communities, the structure and distribution of microbial communities in different fillers were determined. Table 3 shows the Alpha diversity indices of A1, A2 and A3, with a Coverage index of 100% indicating that high-throughput sequencing provided reliable data for the analysis of biodiversity. Chao and Ace indices were used to evaluate community richness,^29^ with the established Chao and Ace index values being 871.89 and 880.78 in the s-Fe^0^ system, 1012.05 and 1017.29 in the s-Fe^0^/BC system and 1241.09 and 1198.29 in the s-Fe^0^/BC/MS system, indicating that ICME effectively improved microbial richness in the system, with MnO_2_ further improving the microbial richness. The Shannon index provides a comprehensive evaluation of microbial community richness and uniformity,^30^ with Shannon index values for A1, A2 and A3 of 3.57, 3.67 and 4.47, indicating that ICME effectively promoted species richness and uniformity within the system, which is consistent with the conclusion that ICME can improve microbial diversity in activated sludge. After the addition of manganese sand, Shannon index values increased significantly, confirming that MnO_2_ promoted the richness and uniformity of microbial species within the system. The porous structure and good adsorption performance of manganese sand make it easier for microorganisms to attach and grow on its surface, thus increasing the diversity and quantity of microbial populations. The addition of manganese sand may make the system environment more conducive to the growth of some specific microorganisms, while inhibiting the reproduction of other microorganisms, thus leading to the change of microbial population structure. Meanwhile. These findings show that combining the ICME process with MnO_2_ catalysis leads to the generation of more Fe ions, which is beneficial to the improvement of species richness and uniformity within the system. This supports the previous findings of Guangsheng Qian *et al.*,^31^ where it was shown that Fe ions are beneficial to increasing the number of microorganisms within reactor systems.

**Table 3:** Alpha diversity index values

Sample number	A (C/N = 3 : 1)		
	A1	A2	A3
Chao	871.89	1012.05	1241.09
Ace	880.78	1017.29	1198.29
Shannon	3.57	3.67	4.47
Coverage	1.00	1.00	1.00

#### 3.4.2 Microbial population similarity analysis

**Fig. 8:**
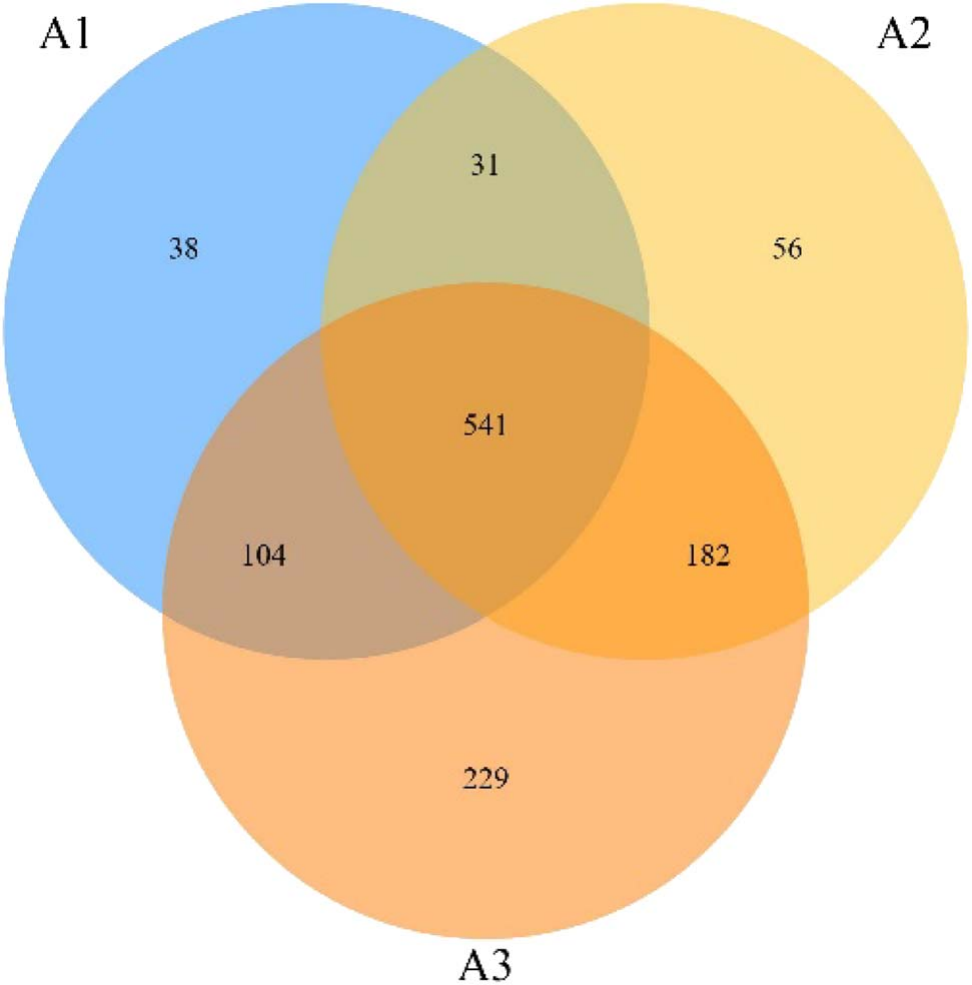
Venn diagram of biological samples from the three reactor groups containing different filler materials.

In order to compare the similarity of microbial populations in different filler material combinations, similarity analysis was performed on microbial populations in the three reactor systems and the results are shown in Fig. 8. The OTU numbers of A1, A2 and A3 were 714, 810 and 1056, respectively, indicating that the ICME system could improve microbial richness, while MnO_2_ further improved microbial richness. The number of OTUs shared by A1 and A2, A1 and A3, and A2 and A3 were 572, 645 and 723, respectively. The similarity of A1 and A2 was 60%, 57.3% and 63.2% according to the Jaccard similarity index. Among the three compared systems, it can be seen that both ICME and MnO_2_ lead to significant changes in the microbial community structure. Although the similarity between microbial communities in A2 and A3 were the highest, the addition of MnO_2_ still resulted in significant changes to the microbial community structure of the ICME system and the successive species changes were analyzed comprehensively.

#### 3.4.3 Microbial species succession analysis

**Fig. 9:**
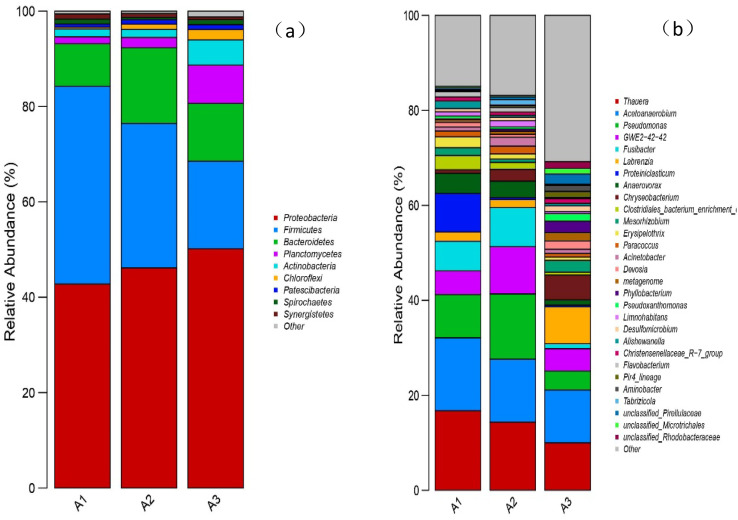
(a) Relative abundance of bacteria at the phylum level for the three reactor groups; (b) relative abundance of bacteria at the genus level for the three reactor groups.

In order to study the influence of different filler combinations on microbial community structure succession, the bacteria in different reactor groups were identified and analyzed at the Phylum level and the genus level. At the phylum level (see Fig. 9(a)), the abundance of *Proteobacteria* and *Firmicutes* in all three groups exceeded 15%. *Proteobacteria* and *Firmicutes* are established as bacteria closely related to denitrification.^22^*Proteobacteria* was considered the dominant phylum in all three reactor groups, with a maximum abundance in A3 of 50.16%, in A2 of 46.15% and in A1 of 42.79%. *Proteobacteria* are Gram-negative bacteria, which contain both autotrophic and heterotrophic bacteria and plays an extremely key role in the decomposition of organic matter and the removal of nitrogen.^29,32^ The observed increase in Proteobacterial abundance may be due to the role of ICME and MnO_2_ catalysis, providing more electron donors for denitrifying microorganisms and strengthening the coupling between microbes and the filler. The relative abundance of *Firmicutes* in A1, A2 and A3 was 41.47%, 30.3% and 18.37%, respectively. Cladosporium are Gram-positive bacteria, which play an important role in the degradation of organic matter, among which the heterotrophic bacteria Bacillus has denitrification capabilities.^33^ It may be speculated that the ICME process with MnO_2_ catalysis may inhibit the growth of heterotrophic denitrifying bacteria. The relative abundances of *Chloroflexi* in the three reactors were 0%, 1.11% and 2.18%. Sorokin *et al.*^34^ first discovered the autotrophic strain Nitrorolancea Holland and Icalbt (Thermomicobia) in a nitrification reactor. Therefore, it may be speculated that the abundance of *Chloroflexi* is closely related to autotrophic denitrification and that ICME and MnO_2_ catalysis can improve the abundance of autotrophic denitrification bacteria. Shihai Deng *et al.*^35^ found that Fe^2+^ and [H] produced by ICME can provide electrons for autotrophic denitrifying bacteria, while Ningfan Song *et al.*^26^ found that ICME accelerated electron transfer and promoted autotrophic denitrification. Therefore, it can be inferred from the above data that ICME can provide electrons for denitrifying bacteria and promote autotrophic denitrification, while MnO_2_ catalysis can further accelerate the electron transfer in the system and promote autotrophic denitrification.

At the genus level (Fig. 9(b)), it was found that denitrifying bacteria were dominant in all samples, among which *Thauera* and *Acetoanaerobium* were the main genera, with high relative abundances. In A1, A2 and A3 samples, the abundances of *Thauera* were 16.76%, 14.35% and 10.01%, respectively, while the abundances of *Acetoanaerobium* were 15.36%, 13.27% and 11.11%, respectively. The ratios in samples A2 and A3 were less than in A1, indicating that the addition of biochar and manganese sand inhibited the growth of these two bacteria under the conditions of a sufficient external organic carbon source. Furthermore, the ratio of A3 was less than A2, showing that MnO_2_ further inhibited the growth of these two genera. Zichun Yan *et al.*^25^ found that high concentrations of Mn^2+^ were not conducive to the growth and reproduction of microorganisms such as *Thauera*. Junfeng Su *et al.*^36^ reported that *Thauera* belong to facultative autotrophic denitrifying bacteria, which can use organic carbon sources for heterotrophic denitrification when the organic carbon source is sufficient and can use Fe^2+^, [H] and sulfide for autotrophic denitrification when the organic carbon source is insufficient. Peng Li *et al.*^37^ identified the facultative autotrophic denitrifying bacterium *Acetoanaerobium* in a hydrogen nutrient denitrification reactor, showing that acetate can be produced by H_2_ and CO_2_ for autotrophic denitrification. The present study also identified some bacteria that only existed in A3, such as *Aminobacter*^38^ and *Pseudoxanthomonas*,^39^ further verifying that the addition of manganese sand can improve species diversity within the system.

#### 3.4.4 FAPROTAX function prediction

**Fig. 10:**
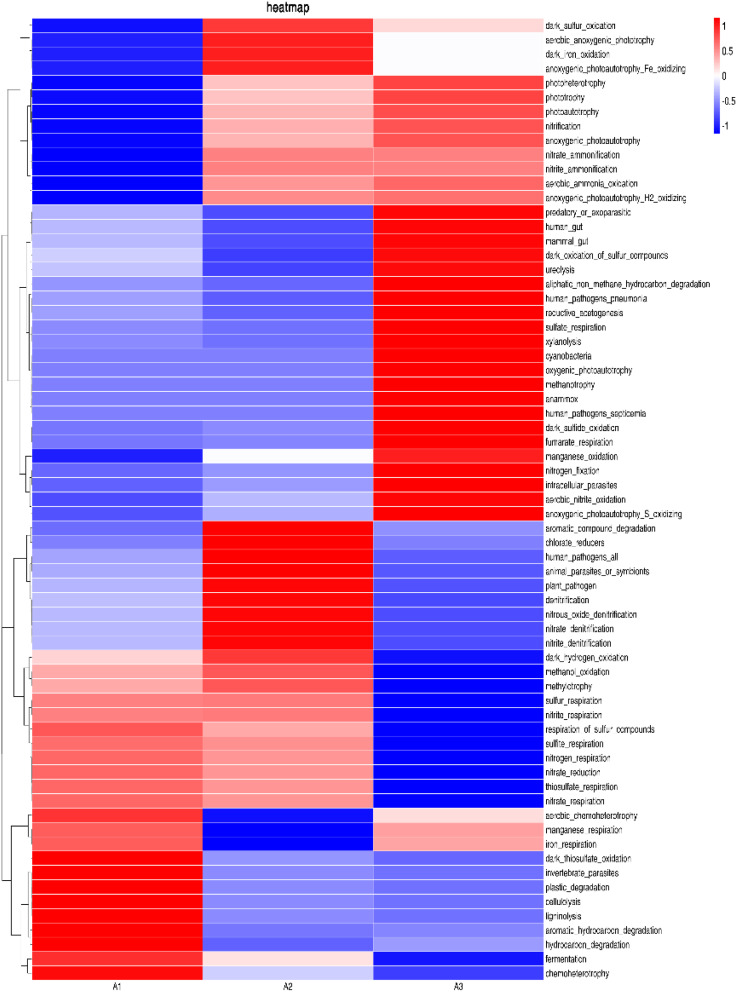
Thermal map of FAPROTAX functional diversity in ICME systems containing different filler combinations.

FAPROTAX was used to annotate the function of bacterial communities in three reactor system groups and 67 functional groups were obtained. As shown in Fig. 10, experimental data shows that the expression levels of chemoheterotrophy in s-Fe^0^, s-Fe^0^/BC and s-Fe^0^/BC/MS systems were 43 316 OTU, 37 289 OTU and 34 205 OTU, respectively, while the nitrogen respiration expression levels were 16 328 OTU, 15 493 OTU and 9154 OTU, the expression levels of nitrate-respiration were 16 230 OTU, 15 483 OTU and 9149 OTU respectively. According to the expression of nitrate respiration and nitrogen respiration in the s-Fe^0^/BC and s-Fe^0^/BC/MS systems, it may be inferred that the addition of manganese sand reduced the activity of microorganisms, although manganese sand can provide a growth environment for some specific microorganisms, it may also inhibit the activity or growth of other microorganisms directly related to nitrate respiration and nitrogen respiration. The number and activity of key microorganisms responsible for nitrate respiration and nitrogen respiration decrease, and the related expression will also decrease, which will affect the heterotrophic denitrification in the system. This is consistent with the previous analysis conclusion that the abundance of Taurea and Anaerobic Acetobacter decreased at the genus level. By comparing the three systems, it was found that the expression of chemoheterotrophy in A1 was significantly higher than in the other two systems and that the ecological function of bacteria in all three systems were mainly nitrate respiration and nitrogen respiration. Nitrate respiration and nitrogen respiration are two ecosystem functions that are performed by most microorganisms and are considered to be two of the most widely extensive functions. Microbial diversity can reflect the stability of community function under changing environmental conditions.^40^ However, FAPROTAX predicts the function based on an artificially constructed database and therefore, its prediction results largely depend on the integrity and accuracy of the database. If the functional information of some bacteria is lacking within the database, or the functional information for known bacteria is inaccurate, it will affect the prediction results. Therefore, FAPROTAX has certain limitations, making it necessary to analyze the community functions of bacteria in the three systems in combination with metagenomics.

## 4. Conclusions

These experimental results provide quantitative and qualitative descriptions of nitrate nitrogen transformation and nitrogen removal for biological and abiotic reactions. The main conclusions are as follows:

(1) In abiotic reaction systems, ICME and MnO_2_ catalysis can promote the transformation of Fe^2+^ to Fe^3+^, generating more electrons, delaying sponge iron surface passivation and strengthening the reduction process of nitrate nitrogen. The main reduction product was ammonia nitrogen, with some nitrite nitrogen accumulation.

(2) The transformation of nitrate nitrogen and the removal of total nitrogen were significantly improved by microbial coupling in the s-Fe^0^, s-Fe^0^/BC and s-Fe^0^/BC/MS systems, with the main reduction product being nitrogen and no nitrite nitrogen accumulation.

(3) Fe^2+^ and Fe^3+^ produced by ICME and MnO_2_ catalysis in the s-Fe^0^/BC and s-Fe^0^/BC/MS systems coupled with microorganisms, served as electron shuttles to strengthen the biological denitrification process and effectively couple the manganese-catalyzed ICME reaction and microbial denitrification process.

(4) The species diversity within the s-Fe^0^/BC and s-Fe^0^/BC/MS systems can be effectively improved by ICME and MnO_2_ catalysis. *Thauera* and *Acetoanaerobium* were identified as the main microorganisms in all three systems. MnO_2_ was not conducive to the growth of facultative bacteria *Thauera* and *Acetoanaerobium*, but it could promote the growth of autotrophic denitrifying bacteria in *Chloroflexi*.

The above conclusions provide a scientific basis and a novel solution for overcoming the problem of nitrate nitrogen pollution using the manganese-catalyzed ICME microbially coupled system.

## Conflicts of interest

There are no conflicts to declare.

## Supplementary Material
